# Mice lacking Kcns1 in peripheral neurons show increased basal and neuropathic pain sensitivity

**DOI:** 10.1097/j.pain.0000000000001255

**Published:** 2018-05-24

**Authors:** Christoforos Tsantoulas, Franziska Denk, Massimo Signore, Mohammed A. Nassar, Kensuke Futai, Stephen B. McMahon

**Affiliations:** a Wolfson Centre for Age-Related Diseases, King's College London, London, United Kingdom; b Developmental Biology and Cancer, UCL Great Ormond Street Institute of Child Health, London, United Kingdom; c Department of Biomedical Science, The University of Sheffield, Sheffield, United Kingdom; d Brudnick Neuropsychiatric Research Institute Department of Neurobiology, University of Massachusetts Medical School, Worcester, MA, United States

**Keywords:** Kv channels, Potassium channels, Kv9.1, Kcns1, Neuropathic pain, Peripheral neuropathy, Nerve injury

## Abstract

Voltage-gated potassium (Kv) channels are increasingly recognised as key regulators of nociceptive excitability. Kcns1 is one of the first potassium channels to be associated with neuronal hyperexcitability and mechanical sensitivity in the rat, as well as pain intensity and risk of developing chronic pain in humans. Here, we show that in mice, Kcns1 is predominantly expressed in the cell body and axons of myelinated sensory neurons positive for neurofilament-200, including Aδ-fiber nociceptors and low-threshold Aβ mechanoreceptors. In the spinal cord, Kcns1 was detected in laminae III to V of the dorsal horn where most sensory A fibers terminate, as well as large motoneurons of the ventral horn. To investigate Kcns1 function specifically in the periphery, we generated transgenic mice in which the gene is deleted in all sensory neurons but retained in the central nervous system. *Kcns1* ablation resulted in a modest increase in basal mechanical pain, with no change in thermal pain processing. After neuropathic injury, Kcns1 KO mice exhibited exaggerated mechanical pain responses and hypersensitivity to both noxious and innocuous cold, consistent with increased A-fiber activity. Interestingly, *Kcns1* deletion also improved locomotor performance in the rotarod test, indicative of augmented proprioceptive signalling. Our results suggest that restoring Kcns1 function in the periphery may be of some use in ameliorating mechanical and cold pain in chronic states.

## Abstract 2

Supplemental Digital Content is Available in the Text.

Deletion of the potassium channel Kcns1 from peripheral sensory neurons alters acute and neuropathic pain processing. Compounds that enhance Kcns1 activity may provide analgesia.

## 1. Introduction

Neuropathic pain remains suboptimally treated, with less than half of patients achieving satisfactory pain relief.^10^ Consequently, determining the molecular constituents of pain sensitivity, and in particular genes influencing susceptibility to chronic pain, remains an important endeavour of pain research.^4,44,45,49^

Voltage-gated potassium (Kv) channels have attracted significant interest as regulators of pain due to their fundamental role in shaping nociceptive signalling. Kv activity in the dorsal root ganglion (DRG) limits neuronal excitability and is characteristically reduced in a range of pain syndromes, from traumatic and metabolic neuropathies to autoimmune disorders.^43^ For instance, members of the Kcna (Kv1) family set spike threshold and firing frequency in the DRG, and Kv1 activity is attenuated in rodents subjected to nerve injury and humans with idiopathic pain.^1,15,51^ Kv function can also be altered by inflammation; thus, “de-sensitisation” by inflammatory mediators reduces Kcnq2/3 (Kv7.2/Kv7.3) background (BG) conductance and promotes DRG hyperexcitability.^27^

KCNS1, encoding the Kv9.1 subunit, is the first Kv gene associated with chronic pain development in humans. Thus, genetic analysis has linked KCNS1 polymorphisms to basal pain sensitivity as well as propensity to develop phantom limb pain, back pain, and HIV-associated pain.^7,16,25^ Kcns1 is abundantly expressed in the DRG, spinal cord, and brain, but excluded from nonneuronal tissues such as muscle, heart, lung, kidney, and liver.^36^ In sensory nerves of rats, Kcns1 mRNA is dramatically and rapidly downregulated by nerve injury, with a time course that matches the development of pain phenotypes.^46^ Recapitulating the Kcns1 loss-of-function triggers neuronal hyperexcitability in myelinated sensory neurons and induces mechanical sensitivity in naive rats, suggesting that physiological Kcns1 activity is pain protective.^46^ Interestingly, Kcns1 conduction is reliant on heteromerisation with members of the Kcnb (Kv2) superfamily^36^; studies in heterologous expression systems and simulations indicate that although nonconducting on their own, the association of Kcns with Kcnb members can stabilise the resultant currents and promote closed-state inactivation that attenuates excitability.^23,34,37^ Recent data using ex vivo recordings also support the notion that Kcnb/Kcns1 signalling in sensory neurons is indeed compromised by nerve injury.^47^

Further dissecting the role of Kcns1 is hampered by the lack of pharmacological tools to specifically target this member within the highly conserved Kv family. We have previously used intrathecal siRNAs to overcome this limitation; however, the robust expression of Kcns1 in the nervous system made it impossible to distinguish between peripheral and central effects of the knock-down. To more thoroughly address this, we generated Advillin-driven inducible transgenic mice in which Kcns1 is selectively inactivated in DRG neurons. We phenotypically characterised these mice to assess the effect of peripheral Kcns1 inhibition on a range of acute and neuropathic pain modalities as well as locomotor behaviour.

## 2. Materials and methods

### 2.1. Animals

PCR was used to clone the mouse *Kcns1* gene into a plasmid containing loxP sites and a neomycin selection cassette flanked by 2 frt sites. The resulting construct contained the entire sequence of *Kcns1*, including flanking regions (2.3 kb upstream of exon 1 and 0.95 kb downstream of the last exon 4). The coding region of *Kcns1* is restricted to exon 3, which was inserted between the 2 loxP sites. The final targeting vector was sequenced, linearised using a NotI digest, and electroporated into 129 mouse embryonic stem (ES) cells. Positive clones were identified by Southern blotting and PCR and used to generate live mice. The ES cell manipulations and blastocyst injections were performed by the Transgenic Services of the Institute of Child Health at University College London. After breeding out the neomycin resistance gene from founders using Flp recombinase mice, the mouse line was crossed with AdvCreERT2 BAC transgenics.^26^ The animals are maintained as homozygous floxed for kcns1 and hemizygous for Cre on a mixed 129SvEv and C57BL/6 J BG.

Kcns1 deletion was induced by intraperitoneal injection of 75 mg/kg tamoxifen (Sigma, H7904) at a volume of 10 mL/kg body weight, for 5 consecutive days. Tamoxifen was initially dissolved in ethanol at a concentration of 100 mg/mL, and subsequently diluted in autoclaved sunflower oil to achieve a stock solution of 10 mg/mL according to manufacturer's instructions. A minimum of 10 days was allowed after tamoxifen treatment, before biochemical or behavioural assessment of the knock-out.

Mice were housed in groups of 5 with standard rodent food and water ad libitum, on a 12-hour light/dark cycle and allowed to acclimatize to their environment for at least 1 week before study. All procedures were compliant with the Home Office (United Kingdom) regulations and Animals (Scientific Procedures) Act 1986, as well as guidelines established by King's College London, the Committee for Research and Ethical Issues of IASP.

### 2.2. Behavioural testing

Pain phenotypes were characterized using 8- to 12-week-old mice. All behavioural testing was conducted during the day in a quiet temperature-controlled room by an experimenter blinded to mouse genotype. Acute phenotypes were assessed using either male or female mice, whereas neuropathic pain was assessed using male mice only, as indicated in the relevant Results sections.

### 2.3. Mechanical pain

Mechanical sensitivity was assessed with the automatic von Frey method using a dynamic plantar aesthesiometer (Ugo Basile). Briefly, animals were placed in a ventilated Plexiglass cage on an elevated wire mesh grid and allowed to acclimatize. Once exploratory behaviour had ceased, an actuator filament (0.5 mm diameter) under computer control delivered a 10-second ramp of increasing force (0-5 g) to the plantar surface of the hind paw until the animal withdrew the paw. Paw withdrawal thresholds were averaged over at least 5 measurements with 5-minute intervals between assays.

### 2.4. Noxious heat pain

Thermal sensitivity was evaluated with the Hargreaves method. Mice were placed in a ventilated Plexiglass cage on an elevated glass floor and allowed to acclimatize. A calibrated radiant light source (Ugo Basile) delivered a constant thermal stimulus (190 mW/cm^2^) to the plantar hind paw, and the latency until withdrawal was recorded. Withdrawal thresholds were averaged from at least 5 consecutive tests, with a minimum of 5 minutes in between measurements. A cutoff of 25 seconds was imposed to prevent tissue damage.

### 2.5. Cold pain

Paw sensitivity to noxious cold was measured by placing mice on a cold plate (Harvard Apparatus) set at 0°C and counting the number of paw lifts within a 2-minute period. To determine sensitivity to a range of cold temperatures (both innocuous and noxious) after nerve injury, mice were subjected to a 2°C/minute cooling ramp from 20°C to 0°C, as previously described.^50^ Paw lifts of the ipsilateral side only and the temperature at which they occurred were recorded and grouped into 2°C bins for analysis. Animals were removed from the plate 2 minutes after the temperature reached 0°C to prevent tissue injury.

### 2.6. Rotarod

Mice were placed on a moving rotarod (Ugo Basile) programmed to accelerate from 4 to 40 rpm, over 120 seconds. After a 3-day training period, the time the mice managed to remain on the rotarod was averaged over 3 consecutive trials. Falls due to poor placing by the experimenter (typically before 5 seconds had elapsed on the rotarod) were excluded, and the trial repeated.

Mechanical, noxious heat pain, and cold pain thresholds were measured sequentially on the same day, with at least a 2-hour interval in between tests to avoid sensitization of the nociceptive system, whereas locomotor behavior was assessed on a separate day.

### 2.7. Neuropathic pain model

Animals were anesthetized with isoflurane (Abbott Animal Health, United Kingdom), and the left hind paw was shaved and sterilized. The sciatic nerve was carefully exposed and isolated from neighboring connective tissue through a small incision midway of the left thigh. A 5.0 vicryl suture (Ethicon) was inserted into the nerve distal to the posterior biceps' semitendinosus branch and ligated so that approximately one-third to one-half of the nerve was held tightly. Pain thresholds were assessed at uninduced baseline, after tamoxifen treatment, and days 7, 10, 14, and 21 after injury.

### 2.8. Antibodies

To generate Kcns1 antibody, the C-terminal end region of mouse Kcns1 (aa 469-497, NP_032461.2) was PCR amplified and subcloned into pGEX-4T vector (glutathione S-transferase fusion protein, Amersham Pharmacia Biotech). Glutathione S-transferase fusion proteins were purified using glutathione-Sepharose 4B resin (Amersham Pharmacia Biotech) and used to immunize rabbit. Kcns1 antibody was affinity purified using glutathione S-transferase fusion protein. Primary antibodies and their dilutions used in this study were: rabbit anti-Kcns1 (1:2000), mouse anti-NeuN (1:1000, Abcam #ab104224), chicken anti-β3tubulin (1:1000, Abcam #ab41489), chicken anti-peripherin (1:500, Abcam #ab39374), goat anti-CGRP (1:1000, Abcam #ab36001), chicken anti-NF200 (1:1000, Merck Millipore AB5539), mouse anti-Na_v_1.8 (1:1000, Neuromab N134/12), goat anti-CSF1 (1:1000, R&D Systems #AF416), and rabbit anti-ATF3 (1:500, Santa Cruz Biotechnology #sc-188). Secondary antibodies used were donkey anti-mouse IgG-conjugated Alexa Fluor 488 or 594, goat anti-chicken IgG-conjugated Alexa Fluor 594, donkey anti-rabbit IgG-conjugated Alexa Fluor 488 or 594, and donkey anti-goat–conjugated Alexa Fluor 594 (all 1:1000, Invitrogen). IB4 binding was visualized using biotin-conjugated IB4 (1:200, Sigma L2140) and Avidin-FITC (1:500, Sigma).

### 2.9. Immunohistochemistry

Mice were culled through cervical dislocation; DRG and sciatic nerve were removed and fixed in 4% paraformaldehyde in 0.1 M phosphate buffer (pH 7.4) for either 20 minutes (DRG) or 2 hours (nerve) at 4°C. The spinal cord was removed after transcardial perfusion with 4% paraformaldehyde and fixed for 4 hours. Tissue was equilibrated in 20% sucrose in phosphate-buffered saline (PBS) at 4°C for 24 hours (DRG) or 72 hours (spinal cord and nerve), embedded in optimum cutting temperature (O.C.T., VWR International) compound and rapidly frozen on liquid N_2_. Tissue sections were cut at the required thickness (DRG, 10 μm; spinal cord/nerve, 20 μm) on a cryostat and thaw mounted onto Superfrost Plus glass slides (VWR). Before antibody staining, nonspecific immunoreactivity (IR) was blocked by 1-hour incubation in PBS supplemented with 0.3% Triton X-100 (Sigma), 4% donkey serum (Jackson ImmunoResearch Labs), and 0.5% skimmed milk (Tesco).

Slides were washed in PBS and incubated overnight at 4°C with the primary antibody in staining buffer solution (PBS supplemented with 0.3% Triton X-100). The following day, slides were washed and incubated for 2 hours with staining buffer containing the appropriate secondary antibodies. After a final wash, slides were mounted with FluorSave mounting medium (Calbiochem).

### 2.10. Image analysis

Antibody staining was visualized on a fluorescence microscope (Zeiss), and images were acquired using a digital camera running Axiovision software. For quantification of Kcns1 immunoreactivity (IR), staining was performed at the same time across all samples to minimize experimental variability, and the same exposure settings were used for image acquisition. Analysis of Kcns1 signal was performed with ImageJ software (http://rsbweb.nih.gov/ij/). For each image, 3 background (BG) measurements were taken, and the mean intensity of BG as well as standard error (SEM) was calculated; a cell was only considered positive when the intensity of its IR was higher than 2xBG + 2xSEM. These objective criteria correlated well with subjective criteria of positively labelled cells. Measurements were conducted using images taken with a 25× objective, only on cells that had a clearly visible nucleus. Between 100 and 200 DRG neurons and 50 to 100 motoneurons per animal were quantified.

### 2.11. Western blot

Full-length kcns1 cDNA was PCR amplified from mRNA isolated from hippocampal primary neurons and cloned in the pCAG-EGFP vector. The shRNA vector targeting kcns1 was purchased from Dharmacon (V2LMM_218480). For immunoblotting, HEK 293T cells were transfected with Kcns1 with or without Kcns1 shRNA using lipofectamine 2000 (Invitrogen). Protein lysates were harvested 3 days after transfection, and immunoblotting was performed using custom-made rabbit anti-kcns1 antibody (1 μg/μL) followed by HRP-conjugated anti-rabbit IgG antibody (GE Healthcare, Little Chalfont, United Kingdom, 1:2000) and Western Lightning Plus-ECL (Perkin Elmer, Waltham, MA).

### 2.12. Quantitative real-time PCR

Mice were killed with an overdose of pentobarbital, perfused with 10 mL PBS and their lumbar DRG dissected and snap frozen in liquid nitrogen. RNA was extracted using a hybrid method of phenol extraction (TRIzol; Invitrogen Carlsbad, CA) and column purification (RNeasy Microkit; Qiagen, Hilden, Germany), including DNase treatment to prevent genomic contamination. After purification, the RNA was eluted with RNase-free water, and its concentration and purity estimated with a NanoDrop ND-100 spectrophotometer (Thermo Fisher Scientific). First strand cDNA was reverse transcribed using Superscript II Reverse Transcriptase, reaction buffer, DTT (all from Invitrogen), random primers, and dNTP mix (Promega), according to the manufacturer's guidelines. Three different sets of exon-spanning primers were used to quantify *Kcns1* levels in DRG using standard qRT-PCR: ex1-2: 5′F-tggagaccaatacagttgtaggtaa, 5′R-tggacaagcttggatttggga; ex2-3: 5′F-gagccaccgtggacatttgg, 5′R-acgacagtgtgcctcccttg; ex3-4: 5′F-tagtgcaagtgttccgcctc, 5′R-cctcacggtagctgtgcttg. All primers were assessed for their efficiency and specificity, and the ddCT method was used with ywhaz (5′F-agtcgtacaaagacagcacgctaa, 5′R-aggcagacaaaggttggaagg) and gapdh (5′f-aggtcggtgtgaacggatttg, 5′r-tgtagaccatgtagttgaggtca) as housekeepers.

### 2.13. Statistical analysis

All data are presented as mean ± SEM, and group sizes are noted in each figure legend. All replicates refer to biological replicates. Statistical significance was determined using GraphPad Prism version 6.0 (La Jolla) using the unpaired, equal variance, 2-tailed Student *t* test, 1-way or 2-way analysis of variance, and Tukey post hoc, as indicated in the corresponding figure legends. All significance tests were justified as appropriate given the study design and nature of comparisons. Significance levels are indicated by: *P < 0.05; **P < 0.01; and ***P < 0.001. A *P* < 0.05 was considered significant.

## 3. Results

### 3.1. Kcns1 expression in sensory neurons

We first investigated Kcns1 expression in the mouse DRG using Kcns1 immunohistochemistry. Kcns1 protein was detected in approximately 40% of all neurons, identified by their IR to β3tubulin (Suppl. Fig. 1A, available online at http://links.lww.com/PAIN/A575). The majority of Kcns1 signal was localised in medium–large diameter neurons that also stained for neurofilament-200 (NF200). Thus, cell size distribution analysis revealed that 40.8% of medium and 69.2% of large diameter neurons expressed Kcns1. By contrast, very little expression was found in small nociceptive neurons, distinguished by peripherin labelling (Suppl. Fig. 1B, available online at http://links.lww.com/PAIN/A575). Accordingly, Kcns1 expression showed very limited overlap with that of calcitonin gene-related peptide (CGRP) and isolectin B4 (IB4) in peptidergic and nonpeptidergic nociceptors, respectively, with the notable exception of some larger neurons positive for CGRP. This expression profile suggests that in the mouse, Kcns1 is enriched in myelinated A fibers, including nociceptive (ie, Aδ and a minority of Aβ) and nonnociceptive (ie, Aβ low-threshold mechanoreceptors) afferents, in agreement with our previous report on Kcns1 expression in the rat.

**Figure 1.**
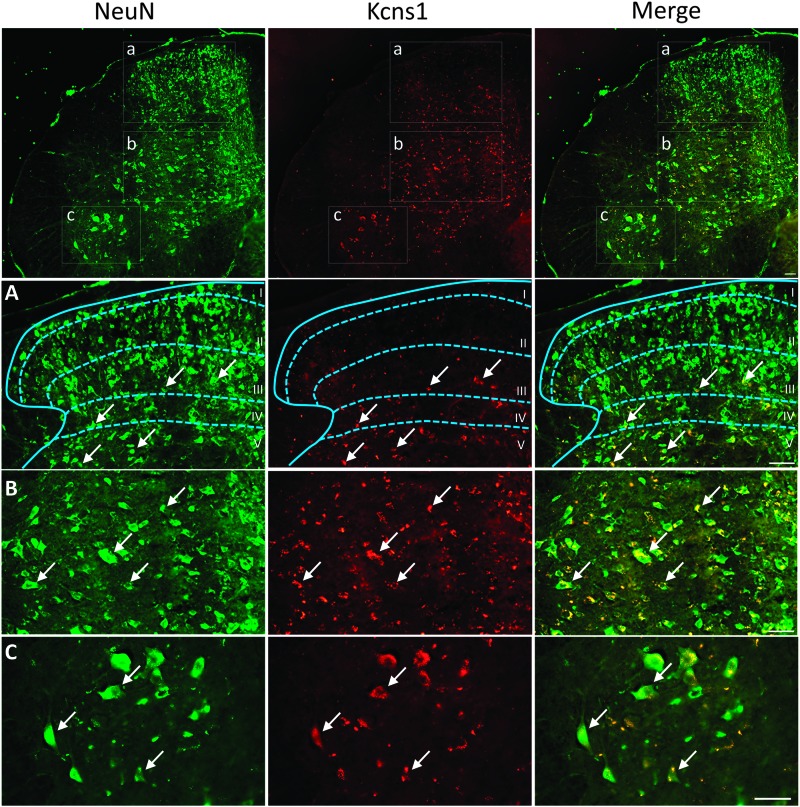
Kcns1 expression in the mouse spinal cord. In the lumbar spinal cord, Kcns1 signal was detected in deeper laminae of the dorsal horn, where A fibers terminate. Thus, very little Kcns1 staining was observed in laminae I to II, but many immunoreactive neurons could be seen in laminae III to V (arrows in magnified inset a) and deeper laminae V to VI (inset b). Kcns1 labelling was also evident in large motoneurons of the ventral horn (inset c). Kcns1 appeared restricted to neuronal cells, as indicated by colocalisation with NeuN and the absence of staining in the white matter. Observations from n = 4 mice. Scale bars = 100 μm.

In the spinal cord, Kcns1 IR was more pronounced in neurons of laminae III to V of the dorsal horn as well as larger neurons in deeper laminae VI and VII. By contrast, very little Kcns1 staining was detected in laminae I to II, where small nociceptors terminate. In addition, Kcns1 signal was particularly prominent in large motor neurons of the ventral horn (Fig. 1). This expression pattern is consistent with the selective expression of Kcns1 in A fibers, most of which project to laminae III to V, with the sporadic staining in lamina I most likely corresponding to Aδ-fiber terminals.

**Figure 2.**
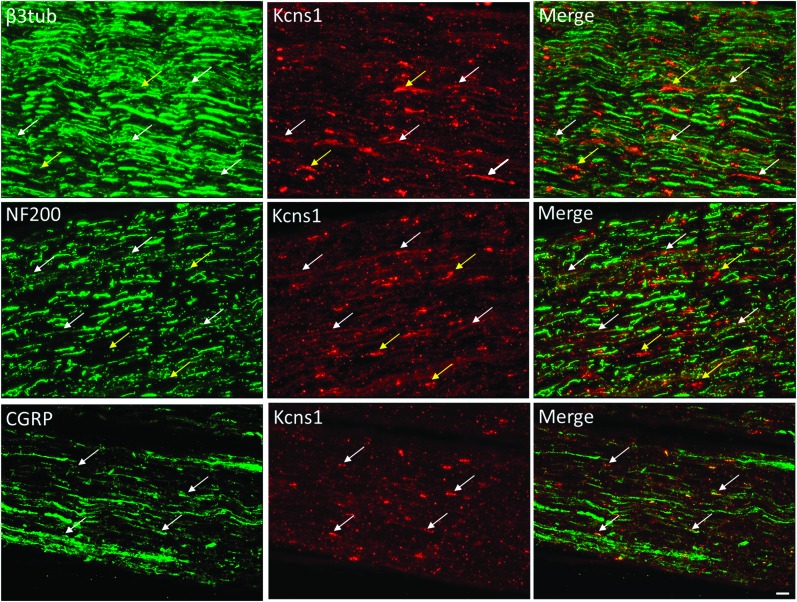
Kcns1 expression in the peripheral nerve. Kcns1 was detected along myelinated nerve fibers of the sciatic nerve, indicated by colocalisation with β3tubulin (white arrows). The Kcns1-expressing fibers were also found to express NF200 and CGRP, in agreement with the documented Kcns1 expression profile in the DRG. Some of the Kcns1-IR appeared to be nonneuronal (yellow arrows). Observations from n = 4 mice. Scale bars = 40 μm. CGRP, calcitonin gene-related peptide; DRG, dorsal root ganglion; IR, immunoreactivity.

We next examined Kcns1 expression in the peripheral nerve. In line with expression of this protein in medium–large DRG neurons, Kcns1 IR could be seen along many NF200-positive, but also some of the larger CGRP-positive fibers of the sciatic nerve (white arrows in Fig. 2). Some of the Kcns1 signal was retained in tissue from KO mice and did not colocalise with β3tubulin (Suppl. Fig. 2, available online at http://links.lww.com/PAIN/A575), suggesting nonneuronal IR. Omitting the primary antibody gave a similar pattern (Suppl. Fig. 3, available online at http://links.lww.com/PAIN/A575), suggesting that this residual signal is due to tissue autofluorescence and/or nonspecific IR of the secondary antibody. In agreement with this, RNA sequencing of Schwann cells isolated from the rat sciatic nerve indicates that Kcns1 transcript is absent in both naive and injured (nerve crush) conditions (Ref. 3 and personal communication with Brosius Lutz).

### 3.2. Generation of Kcns1 transgenic mice

**Figure 3.**
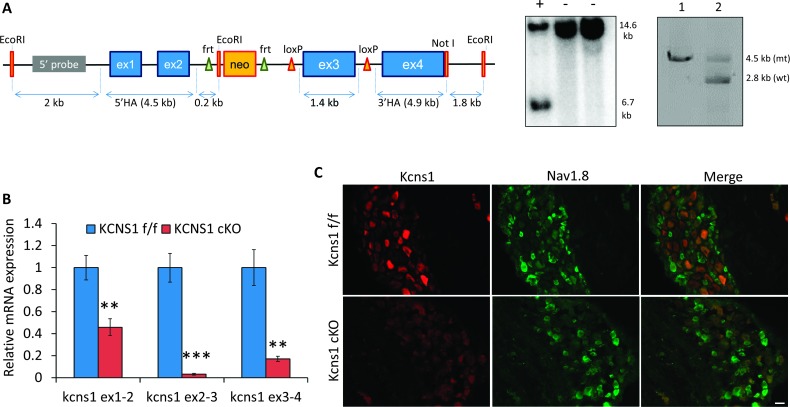
Generation of Adv-CRE inducible Kcns1 conditional KO (cKO) mice. (A) Floxed *Kcns1* knock-in mice were generated using homologous recombination in ES cells and subsequent blastocyst injections. (A) Schematic of the targeting construct is displayed here. Successful clones were identified using Southern blotting (middle, +: mutant clone) and PCR (right, 1: original targeting plasmid and 2: successful ES cell clone). The neomycin cassette was removed using an flp enzyme, and the resulting *Kcns1* mice were crossed with the AdvCreERT2 line to generate an inducible knock-out of the *Kcns1* gene in sensory and sympathetic neurons. (B) After induction of gene knock-out through tamoxifen treatment, DRG from cKO mice and floxed controls were analysed for Kcns1 mRNA expression by qPCR. Using primers amplifying within the deleted region of exon 3 (primer pairs 2-3 and 3-4), we found 97% and 89% reductions of Kcns1 mRNA in cKO mice compared with floxed controls. Using primers that bind before the targeted exon (exons 1-2), we also observed a 54% reduction in Kcns1 transcripts, most likely indicating degradation of incompletely formed mRNAs (**P<0.01, ***P<0.001; n = 3/group, the Student *t* test). (C) Kcns1 transcript reduction in cKO mice was also reflected at the protein level, as revealed by Kcns1 immunoreactivity in the DRG after tamoxifen treatment. As expected, Kcns1 protein was detected in medium–large, predominantly Na_v_1.8-negative neurons of floxed controls but was virtually absent from cKO mice. Observations from n = 3 mice/group. Scale bar = 40 μm. DRG, dorsal root ganglion; ES, embryonic stem; qPCR, quantitative real-time PCR.

Having characterized its expression in the DRG, we sought to genetically silence Kcns1, specifically in the peripheral nervous system. To this end, we used tamoxifen-induced Cre recombination under the control of the Advillin promoter to delete the *Kcns1* gene in all sensory neurons. Floxed *Kcns1* knock-in mice were generated by blastocyst injection (Fig. 3A), and crossed with existing AdvCreERT2 BAC transgenic mice^26^ to allow for spatially and temporally controlled deletion of *Kcns1*.

Quantification of Kcns1 mRNA by quantitative real-time PCR after tamoxifen treatment confirmed a large reduction in mRNA levels of Kcns1 cKO mice compared with floxed littermates (Kcns1 f/f) (Fig. 3B). We used 3 pairs of exon-spanning primers targeting all exons of the gene, including the floxed coding exon 3. As expected by the design of the transgenic line, amplification with primers binding within exon 3 indicated large mRNA reductions in cKO mice (primers 2-3 and 3-4, reductions of 97% and 89% compared with Kcns1 f/f, *P* < 0.001 and *P* = 0.002, respectively). Amplifying the two 3′ untranslated regions preceding the targeted exon (primers 1-2) also demonstrated a 54% reduction, presumably due to degradation of incompletely formed Kcns1 mRNAs (*P* = 0.007).

Next, we confirmed that this mRNA reduction resulted in loss of the translated protein, by examining Kcns1 IR in DRG neurons after tamoxifen induction (Fig. 3C). In the control littermates, Kcns1 IR was observed in medium–large neurons, the majority of which are negative for the nociceptive neuron marker Na_v_1.8. In cKO mice, however, Kcns1 staining was virtually absent from both small and medium–large neurons, confirming deletion at the protein level. To confirm that gene deletion is restricted to peripheral neurons, we also examined Kcns1 expression in large motoneurons of the spinal cord in tamoxifen-induced control and KO mice (Suppl. Fig. 4, available online at http://links.lww.com/PAIN/A575). Kcns1 IR was high in both groups, expressed in more than 90% of motoneurons with no statistical difference between genotypes (93.5 ± 1.1% vs 91.9 ± 0.7% for floxed and cKO, respectively, *P* = 0.253). Quantification of denaturated protein levels using western blot was not feasible as the antibody produced nonspecific results in this application (Suppl. Fig. 5, available online at http://links.lww.com/PAIN/A575).

### 3.3. Acute pain processing in Kcns1 cKO mice

**Figure 4.**
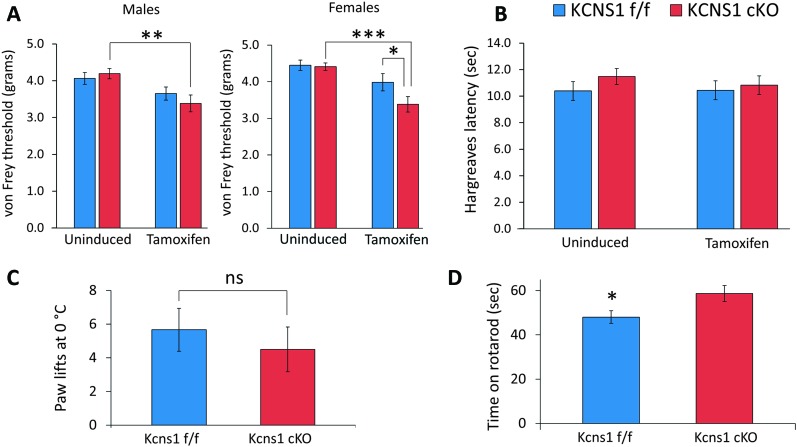
Kcns1 deletion from sensory neurons affects basal pain processing. (A) Sensitivity to mechanical stimulation of the hind paw in conditional KO (Kcns1 cKO) and littermate control (Kcns1 f/f) male (left) and female (right) mice. Before induction of gene deletion with tamoxifen (“uninduced”), there is no difference in responses between genotypes (*P* > 0.05, the Student *t* test). However, 10 days after induction, there is a ∼20% decrease in pain thresholds in cKO mice compared with preinduced baseline (*P<0.05, **P<0.01, ***P<0.001; n = 11 male mice and n = 12 female mice/group; 2-way repeated-measures ANOVA with Tukey) (see also Suppl. Fig. 6, available online at http://links.lww.com/PAIN/A575). (B) Responses to noxious heat were not affected by Kcns1 deletion, as assessed with the Hargreaves method 10 days post-tamoxifen (*P* > 0.05; n = 11 male mice/group; 2-way repeated-measures ANOVA). (C) Kcns1 cKO mice and floxed littermates exhibited similar responses to noxious cold stimulation when placed on a 0°C cold plate (*P* > 0.05; n = 12 female mice/group; the Student *t* test). (D) Kcns1 deletion from sensory neurons resulted in enhanced locomotor performance in the rotarod test (*P<0.05; n = 12 female mice/group; the Student *t* test). ANOVA, analysis of variance.

To characterize the effect of peripheral Kcns1 deletion on acute pain, we compared mechanical thresholds of male Kcns1 cKO mice and Kcns1 f/f littermates (Fig. 4A, left graph), using the automatic von Frey method. Before tamoxifen induction (“uninduced”), both genotypes showed similar responses to stimulation (4.2 ± 0.1 vs 4.1 ± 0.2 g, respectively; *P* > 0.05). After gene induction, however (10 days post-tamoxifen), there was an approximately 20% reduction of mechanical thresholds in the cKO group (down to 3.4 ± 0.2 g, *P* < 0.01 vs uninduced cKO baseline). By contrast, no significant reduction was found in the f/f littermates after tamoxifen induction (*P* > 0.05 vs uninduced f/f baseline). This result suggests an effect of gene knock-out, although the difference between genotypes after tamoxifen did not reach significance (*P* > 0.05; tamoxifen f/f vs tamoxifen cKO). A more pronounced effect was observed in female mice (Fig. 4A, right graph); gene deletion induced a 20% decrease in cKO thresholds of female mice (from 4.4 ± 0.1 to 3.4 ± 0.2 g; *P* < 0.001), which was also 15% lower than the corresponding values in the f/f group after tamoxifen (4.0 ± 0.2; *P* < 0.05). Pooling male and female data together also resulted in a significant reduction of mechanical thresholds when comparing genotypes after tamoxifen (Suppl. Fig. 6, available online at http://links.lww.com/PAIN/A575; 3.8 ± 0.2 vs 3.4 ± 0.2; *P* < 0.05), reinforcing the idea that Kcns1 deletion triggers mechanical hypersensitivity. Pooling the data also revealed a statistically significant difference between uninduced and treated conditions for each genotype (*P* < 0.001 for Cre and *P* < 0.05 for floxed), likely indicating a toxicity-induced effect by tamoxifen.

When assessing noxious heat thresholds after tamoxifen using the Hargreaves method, we found no difference between genotypes (Kcns1 f/f, 10.4 ± 0.7 seconds vs Kcns1 cKO, 10.8 ± 0.4 seconds; *P* > 0.05), suggesting that Kcns1 is not involved in determining basal heat pain (Fig. 4B). In addition, tamoxifen induction did not affect responses to noxious cold (Fig. 4C); thus, Kcns1 f/f and Kcns1 cKO mice exhibited similar numbers of paw lifts on average when placed on a cold plate at 0°C (5.7 ± 1.3 vs 4.5 ± 1.3 lifts, respectively; *P* = 0.53). Together, these data indicate that *Kcns1* deletion causes a modest increase in mechanical pain sensitivity but does not affect basal thermal processing.

Interestingly, when investigating locomotor behavior (Fig. 4D), we found that Kcns1 cKO mice performed better on the rotarod test compared with floxed littermates (58.7 ± 3.6 vs 48.1 ± 2.9 seconds, respectively; *P* = 0.03). This result suggests that Kcns1 deletion in the DRG may enhance excitability of proprioceptive neurons, leading to increased locomotor activity.

### 3.4. Neuropathic pain responses of Kcns1 cKO mice

**Figure 5.**
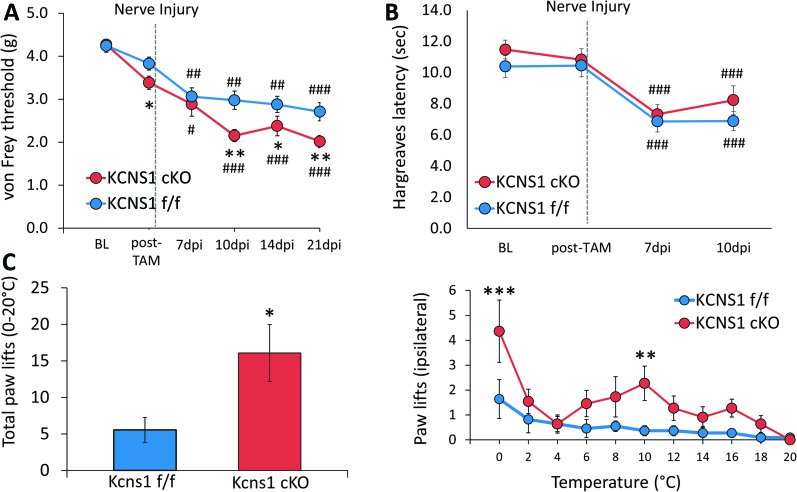
Peripheral Kcns1 deletion triggers exaggerated pain phenotypes after nerve injury. (A) After tamoxifen treatment, the mice were subjected to partial nerve ligation and pain phenotypes monitored thereafter. Nerve injury resulted in pain hypersensitivity in both genotypes lasting for 3 weeks following the insult (#*P* < 0.05 vs post-TAM). In addition, pain thresholds in the cKO group were 27% lower compared with the floxed group at day 10 (**P < 0.01) and remained significantly lower thereafter (n = 11 male mice/group; 2-way repeated-measures ANOVA with Tukey). BL, pretamoxifen baseline; post-TAM, post-tamoxifen; dpi, days post injury. (B) Nerve damage decreased heat pain thresholds (###*P* < 0.001 vs post-TAM), but there was no difference between genotypes (n = 11 male mice/group; 2-way repeated-measures ANOVA). BL, pretamoxifen baseline; post-TAM, post-tamoxifen; dpi, days post injury. (C) Kcns1 deletion also affected behavioural responses to innocuous and noxious cold temperatures at 10 days after injury, manifested as increased paw lifting during a cold temperature ramp from 20 to 0°C. Left, cKO mice showed more paw lifts on average (*P* < 0.05, n = 11 male mice/group; the Student *t* test) during the ramp. Right, distribution of responses (*P* < 0.05; the Kolmogorov–Smirnov test) demonstrating cold hypersensitivity of cKO mice, particularly around temperatures of 10°C (*P* < 0.01) and 0°C (*P* < 0.001, n = 11/group; 2-way repeated-measures ANOVA with Tukey). ANOVA, analysis of variance; cKO, conditional KO.

We next examined whether Kcns1 deletion in sensory neurons impacts neuropathic pain induced by nerve injury. Tamoxifen-treated Kcns1 f/f and cKO mice (“post-TAM”) were subjected to a partial sciatic nerve ligation (Seltzer model), and pain thresholds were monitored thereafter. Nerve injury reduced mechanical pain thresholds in the Kcns1 f/f group compared with preinjury values, commencing on day 7 and persisting throughout the postoperative period studied (*P* < 0.01 vs post-TAM) (Fig. 5A). Nerve damage also reduced mechanical pain thresholds in cKO mice during the same period (*P* < 0.05 vs post-TAM) and from day 10 onwards, thresholds were significantly lower than the respective ones in the floxed group (*P* < 0.05 vs Kcns1 f/f). More specifically, thresholds of cKO mice were 27% lower than those of f/f mice at 10 days (2.2 ± 0.1 vs 3.9 ± 0.2 g; *P* < 0.01), and the magnitude of this difference was similar at 21 days (2.0 ± 0.1 vs 2.7 ± 0.2 g; 26% difference; *P* < 0.01). These results demonstrate that after neuropathic injury, cKO mice develop greater mechanical hypersensitivity compared with control littermates.

When examining responses to a radiant heat source, we found that both groups developed similar levels of heat pain sensitivity after nerve damage (Fig. 5B). Thus, thresholds to heat stimulation dropped by approximately 30% after injury (f/f, 10.4 ± 0.7 [post-tam] to 6.9 ± 0.7 [7 days] and 6.9 ± 0.6 seconds [10 days]; cKO, 10.8 ± 0.7 [post-tam] to 7.3 ± 0.6 [7 days] and 8.2 ± 0.9 seconds [10 days]), with no significant difference between genotypes at any time point (*P* > 0.05).

We finally investigated how nerve injury impacts responses to cold stimuli in these mice (Fig. 5C). For this, the mice were subjected to a temperature ramp (20-0°C), and responses on the ipsilateral side (day 10 after surgery) were assessed. Quantifying the average number of responses during the whole temperature ramp (left panel) revealed that Kcns1 cKO mice exhibited more paw lifts on average compared with nerve-injured f/f mice (16.1 ± 3.9 vs 5.5 ± 1.7 paw lifts, respectively; *P* = 0.02). Examining the distribution of the responses during the temperature ramp (right panel) confirmed significantly augmented cold hypersensitivity in cKO mice (*P* = 0.012 comparing distributions), with the biggest effect observed around 10°C (*P* < 0.01) and 0°C (*P* < 0.001). These data indicate that peripheral Kcns1 deletion increases sensitivity to both innocuous and noxious cold temperatures after nerve injury.

### 3.5. Neurochemical changes after nerve injury

**Figure 6.**
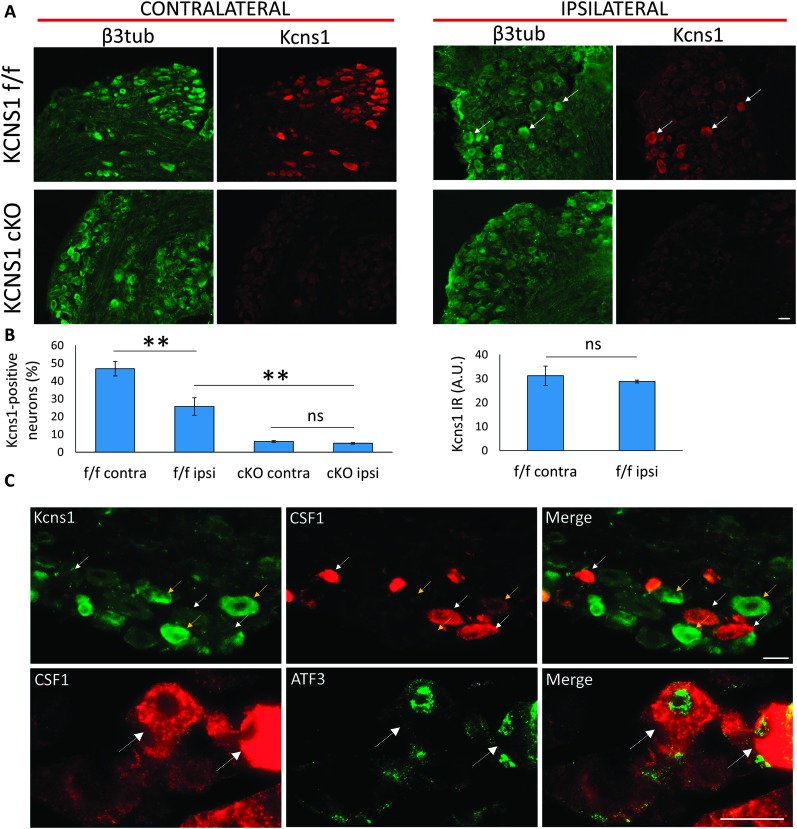
Kcns1 protein expression after nerve injury in cKO and control mice. (A) Kcns1 expression on the contralateral (uninjured) and ipsilateral (injured) DRG from cKO and floxed littermate mice, 21 days after nerve damage. Kcns1 IR is largely attenuated by nerve injury in control mice; however, some Kcns1-positive neurons are still visible (arrows, top row). By contrast, no signal could be observed in either DRG from cKO mice (bottom row). (B) Quantification of percentages of Kcns1-positive neurons (**P < 0.01; n = 3/group; 1-way ANOVA with Tukey) and signal intensity (*P* > 0.05, n = 3/group; the Student *t* test). Scale bar = 40 μm. (C) Downregulation of Kcns1 in injured DRG neurons. Top panels, a fraction of small and medium–large sensory neurons are labelled with the nerve injury marker CSF1 at 21 days post-Seltzer. These injured neurons show no Kcns1 immunoreactivity (white arrows), which is however retained in CSF1-negative neurons (yellow arrows). Bottom panels, CSF1-positive neurons (arrows) show strong nuclear immunoreactivity for the injury marker ATF3. Scale bars, 40 μm. ANOVA, analysis of variance; cKO, conditional KO; DRG, dorsal root ganglion; IR, immunoreactivity.

Previous work in rats has shown that traumatic nerve injury induces a dramatic Kcns1 downregulation at the mRNA level.^46^ To extend these findings and gain more insight into the observed phenotypes in mice, we characterized Kcns1 expression in DRG of f/f and cKO mice at the behavioural end point (21 days post-Seltzer) (Fig. 6A). Strong Kcns1 staining was evident in the contralateral (uninjured) side of the f/f mice; however, Kcns1 signal was substantially attenuated by nerve damage on the ipsilateral side. Quantification of Kcns1 IR showed a significant decrease in the percentage of Kcns1-positive neurons from 46.9 ± 4.0% to 25.6 ± 5.0% (*P* < 0.01) on the injured DRG (Fig. 6B, left). Notably, residual Kcns1 signal remained after nerve damage, most likely corresponding to neurons that were unaffected by the partial nerve injury (two-thirds to a half of all DRG neurons in this model). Indeed, neurons positive for the nerve injury marker colony-stimulating factor 1 (CSF1)^13^ showed no IR for Kcns1, whereas neurons that retained Kcns1 labelling were negative for this marker (Fig. 6C).

Interestingly, when quantifying signal intensity (Fig. 6B, right), there was no difference between ipsi and contra sides (31.2 ± 4.0 vs 28.8 ± 0.6 arbitrary units; *P* = 0.59), suggesting that injured neurons have completely lost Kcns1 protein at this time point, whereas spared neurons retain baseline levels of expression. Finally, as anticipated Kcns1 protein was virtually absent in both ipsilateral and contralateral DRG neurons of cKO mice. This expression profile matches the altered pain responses in cKO and f/f mice after injury and further supports an inversely proportional relationship between reduced Kv9.1 function and magnitude of pain hypersensitivity.

## 4. Discussion

Kcns1 was first implicated in nociception by gene association studies pinpointing protective haplotypes influencing acute pain intensity and neuropathic pain incidence in humans.^7,25^ Further work demonstrated that intrathecal delivery of Kcns1 siRNAs augmented mechanical pain in rats^46^; however, a contribution of Kcns1 knock-down in the central nervous system could not be ruled out. Here, we conclusively demonstrate that targeted Kcns1 deletion in peripheral sensory neurons is sufficient to increase basal mechanical sensitivity, independently of any central Kcns1 function. A second major finding is that peripheral Kcns1 inhibition predisposes towards neuropathic pain, evident by increased mechanical sensitivity in nerve-injured KO mice. Third, we show for the first time that Kcns1 activity is also linked to augmented responses to cold temperatures after neuropathic injury. Finally, we found that Kcns1 knock-out increases locomotive ability, presumably through an increase in proprioceptor excitability.

Kcns1 expression in the mouse was similar to that previously reported in the rat.^46^ Kcns1 protein was predominantly detected in medium–large myelinated neurons and was almost absent from small nociceptive neurons. Kcns1 could also be detected in some medium–large neurons immunoreactive for Na_v_1.8, in agreement with previous estimates that as many as 40% of NF200-positive neurons also express Na_v_1.8.^38^ These most likely represent Aδ and Aβ nociceptors containing pain-signalling neuropeptides such as CGRP and substance P; we indeed observed Kcns1 colocalisation with CGRP in many of these neurons. Away from the cell body, Kcns1 was found along primary afferents extending to the periphery. Many Kv channels occupy juxtaparanodal regions in myelinated neurons, including the Kcns1-interacting subunits Kcnb1 and Kcnb2,^24,44^ and recent data show that Kv complement and organisation in nodes of Ranvier can dynamically change after nerve injury to regulate excitability.^5^ It would therefore be informative to examine nodal assembly of Kcns1 and whether it is affected by nerve lesions in future studies. The high-activation threshold and slow kinetics of Kcnb channels suggest that tetrameric channels with Kcns1 subunits may be progressively recruited during sustained nociceptive stimulation, in contrast to other Kv channels in nerve terminals that can counteract depolarisation rapidly, such as A-type channels.^35^ The diverse complement and topology of Kv channels with distinct biophysical properties may provide sensory neurons with a powerful means to fine-tune nociceptive excitability.

Deletion of Kcns1 from sensory neurons resulted in a modest increase in acute pain, ie, an 8% reduction in mechanical thresholds. This mechanical hypersensitivity is presumably mediated by increased signalling in high-threshold mechanosensitive A fibers (primarily Aδ but could also include Aβ nociceptors), and such A-fiber hyperexcitability has indeed been shown after Kcns1 deletion.^46^ This enhanced nociceptive signalling aligns well with the finding that human KCNS1 polymorphisms are associated with pain sensitivity in healthy volunteers,^7^ as well as pain intensity in patients with breast cancer.^25^ In addition, we show for the first time that peripheral Kcns1 knock-out leads to augmented neuropathic pain phenotypes after nerve injury; this is in agreement with a well-described link between severity of acute pain and propensity for the development of chronic pain in humans, for instance, in the context of persistent postoperative pain.^22^

One area of debate is whether susceptibility to chronic pain is simply the manifestation of such acute pain hypersensitivity in a chronic setting. Our data suggest that there is limited overlap between the two; nerve damage triggered a 27% reduction in mechanical pain thresholds; however, less than a third of this change could be justified by the effect of Kcns1 deletion on acute pain (∼8% reduction). The mechanisms governing the remaining difference are not clear, but a likely explanation is that under chronic pain conditions, Kcns1 works in synergy with injury-induced plasticity, such as inhibition of other Kv channels and/or sensitization of Na_v_ and HCN channels.^44,45,49^ According to one hypothesis, hyperactivity in low-threshold Aβ mechanoreceptors due to Kcns1 dysfunction can mediate mechanical allodynia in neuropathic states due to central sensitisation maintained by C-fiber input. It has been proposed that central sensitisation may even be partly sustained by activity in Aβ fibers that have undergone injury-induced phenotypic switch in their neurochemical complement.^42^ A role of A fibers in chronic pain is supported by the fact that soon after neuropathic injury, the bulk of maladaptive activity is manifested as spontaneous firing in these fibers.^29^ Kcns1 may promote such activity, and we have indeed shown that reduced Kcns1 function triggers spontaneous firing.^46^

Tamoxifen treatment also appeared to have an algogenic effect on mechanical thresholds. This result agrees with recent work highlighting transient toxicity of tamoxifen 4 days after single administration, resulting in cellular stress and induction of nerve injury markers in DRG neurons, particularly proprioceptors.^8^ Although we accessed pain phenotypes 10 days post-treatment, it is possible that some toxicity-induced effects were still present because of the repeated administration regime required for induction of gene KO.

Furthermore, our data expand the role of Kcns1 in nociception by identifying an involvement in cold pain processing. Although Kcns1 deletion did not impact acute detection of noxious cold, we documented a marked sensitivity to both innocuous and noxious cold temperatures in neuropathic conditions. This effect is most likely mediated by Aδ fibers, which can signal a range of cold temperatures,^39^ as illustrated by the complete suppression of cold responses after Aδ-fiber block.^30^ Interestingly, nerve injury increases the proportion of cold-sensitive Aδ fibers,^18^ and it has been proposed that this may be related to removal of an excitability brake normally provided by K^+^ channel conductance.^12,31,32,48^ Kcns1 loss-of-function could act as a similar disinhibition mechanism that promotes conversion of cold-insensitive fibers into cold-sensitive. Although cold sensitivity has also been described in large myelinated afferents (eg, slowly adapting mechanoreceptors such as Merkel disks and Ruffini endings in the skin), these responses are much smaller and therefore unlikely to make a major contribution to cold sensing.^17,19^

Consistent with previous reports in the rat, Kcns1 deletion did not affect responses to noxious heat.^46^ This is expected because heat pain in glabrous skin is encoded by activity in TRPV1-expressing small nociceptive fibers, and our histology indicates that Kcns1 is largely absent from these.^14^ Although Aδ fibers (type II) in hairy skin can signal the “first” sharp pain in response to noxious heat (a component which can be abolished by A block), these afferents are not present in glabrous skin.^33^ Given the existence of a subset of warm-sensitive afferents conducting within the A-fiber range, it would be interesting to investigate whether warm responses are modulated by Kcns1 deletion.^41^

Kcns1 knock-out also improved locomotor function. This is attributed to a loss of Kcns1 function in proprioceptive A fibers (which also express interacting partners Kcnb1 and Kcnb2^47^) because Kcns1 expression in spinal motoneurons was unaltered by the Advillin-driven knock-out. One possible explanation is that loss of Kcns1 promotes excitability in Aα fibers leading to enhanced proprioceptive feedback to muscle spindles, Golgi tendons, and joints and thus improved motor coordination. The robust Kcns1 expression documented in spinal cord motoneurons may be indicative of a more generalised role in motor function, especially because these neurons also express Kcnb subunits.^20^ Interestingly, Kcns1 is also abundantly expressed in the dorsal cochlear nucleus of the cerebellum, an auditory structure that processes incoming proprioceptive information.^11,21^

Our results suggest that restoring Kcns1 activity in the periphery has therapeutic potential in chronic pain. Given the involvement of K^+^ channels in the pathophysiology of divergent pain syndromes, it would be informative to ascertain pain phenotypes of Kcns1 KO mice in experimental paradigms mimicking other pain states. For instance, the cold allodynia that develops during cancer treatment with oxaliplatin is associated with inhibition of K^+^ channels in A fibers, and intraplantar administration of a K^+^ channel blocker can induce cold sensitivity.^9,40^ Painful diabetic neuropathy has been associated with a reduction in sustained delayed K^+^ currents in myelinated DRG neurons,^6^ and Kcnb1 channels are chief determinants of this conductance.^2^ As diabetes does not induce any transcriptional change in Kcnb subunits,^6^ it is possible that altered modulation by Kcns1 subunits underlies the observed electrophysiological phenotype. Finally, it would be interesting to investigate Kcns1 function in models of inflammatory pain, particularly because accumulating data suggest that some Kv channels can be directly modulated by inflammatory mediators.^27,28^

## Conflict of interest statement

The authors have no conflict of interest to declare.

This research was funded by the Wellcome Trust (097903/Z/11/Z to S.B.M.) and National Institutes of Health grants (R01NS085215 to K.F.).

## Appendix A. Supplemental digital content

Supplemental digital content associated with this article can be found online at http://links.lww.com/PAIN/A575.
